# IGF2 loss of imprinting enhances colorectal cancer stem cells pluripotency by promoting tumor autophagy

**DOI:** 10.18632/aging.103837

**Published:** 2020-11-05

**Authors:** Tianyi Gao, Xiangxiang Liu, Bangshun He, Yuqin Pan, Shukui Wang

**Affiliations:** 1 Department of Clinical Laboratory, Nanjing First Hospital, Nanjing Medical University, Nanjing 210006, Jiangsu, China; 2 Central Laboratory, Nanjing First Hospital, Nanjing Medical University, Nanjing 210006, Jiangsu, China; 3 Jiangsu Collaborative Innovation Center on Cancer Personalized Medicine, Nanjing Medical University, Nanjing 210006, Jiangsu, China

**Keywords:** IGF2 LOI, CRC, CSCs pluripotency, autophagy

## Abstract

Cancer stem cells (CSCs) are believed to be the driving force behind the tumor growth. We performed this study to further explore the role of IGF2 epigenetic on CRC stem cells pluripotency which showed that IGF2 LOI CRC cells usually had a higher CD133 expression and sphere forming efficiency than MOI cells. IGF2 LOI CSCs were also found to have a higher level of autophagy than MOI CSCs. Moreover, IGF2/IR-A signal was determined to play a more important role in CSCs formation than IGF2/IGF1R. At last, by using miRNA-195 mimics, we fortunately found the increased IR-A expression might be due to the degradation of miRNA-195 in CRC. In conclusion, our results might reveal that IGF2 LOI could promote CRC stem cells pluripotency by promoting CSCs autophagy. For the degradation of miRNA-195, IGF2 showed a higher ability in interacting with overexpressed IR-A rather than IGF1R which would further activate CSCs autophagy. All these findings might provide a novel mechanistic insight into CRC diagnosis and therapy.

## INTRODUCTION

Colorectal cancer (CRC) is one of the leading causes of cancer-related morbidity and mortality [1]. However, the precise mechanisms and genetic underpinnings of this disease remain to be fully elucidated. Cancer stem cells (CSCs) are a small subpopulation, usually comprise<1% of cancer cells which are believed to be the driving force behind the tumor growth, cancer recurrence, metastasis, and chemoresistance [2, 3]. Targeting these cells, especially their pluripotency of plasticity and self-renewal, represents one of the major efforts in the past two decades to eradicate tumor cells.

Autophagy is an adaptive catabolic process for the preservation of cell homeostasis that stop dividing and enter quiescence, and occurs in response to different forms of stressful conditions, including starvation, hypoxia, and chemo/radiotherapy [4]. Accumulating evidence indicate that the CSCs are in an autophagic state and blockade of autophagy reduces their activity and sensitizes them to antitumor drugs [5]. Moreover, CD133, one of the most commonly used markers for CSCs, was found to promote the autophagocytic activity of hepatoma CSCs [6], suggesting a functional link between CSCs and autophagy. So getting insights into the regulatory factors and the molecular mechanisms by which autophagy exploits its function in CSCs is fundamental for developing more effective and safe antitumor strategies.

Insulin-like growth factor 2 (IGF2) is a 7.5 kDa mitogenic peptide hormone expressed by liver and many other tissues. Dysregulation of differentially methylated region (DMR) on the maternal chromosome causes loss of imprinting (LOI) of IGF2 usually lead to an over-expression and increased sensitivity to IGF2 signal which has been associated with poor prognosis and therapeutic resistance in CRC [7, 8]. Recently, IGF2 was found to play an important role in regulating CSCs stemness [9, 10]. By the way, studies showed that IGF2 could preserve tumor cell survival by creating an autophagic state of dormancy [11, 12]. However, few studies had reported the association between IGF2 and CRC CSCs autophagy.

On general, we perform this study to deep explore the role of IGF2 in CRC stem cells autophagy and its regulation in the maintenance of CSC characteristics hoping to get a better understanding of regulatory factors and their molecular mechanisms in CRC cancer stem cells regulation.

## RESULTS

### The association between IGF2 LOI and CSCs pluripotency

**Figure 1:**
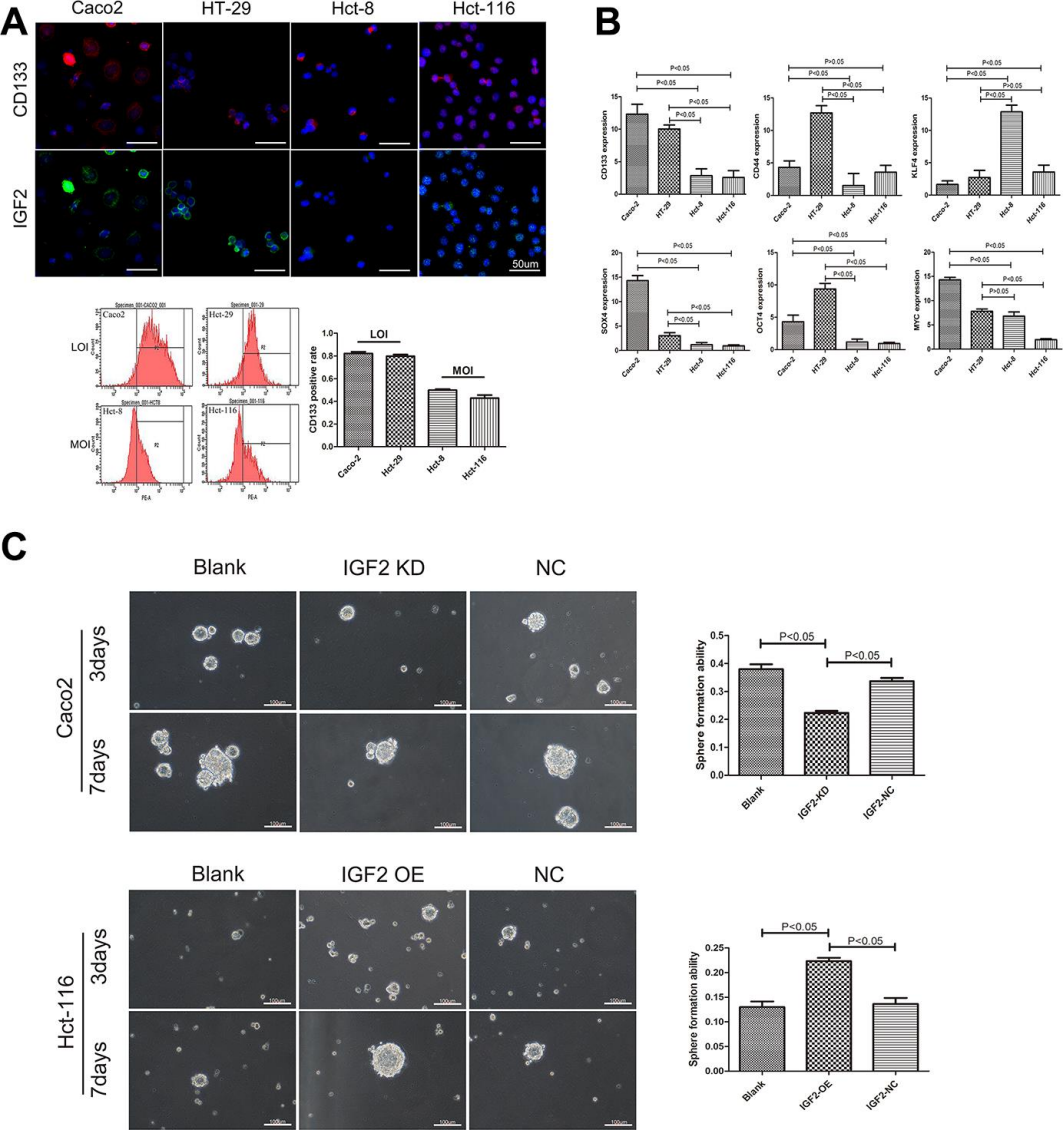
**The association between IGF2 LOI and CSCs characteristics.** (**A**) the correlation between IGF2 LOI and CD133 expression; (**B**) the mRNA expression of CSCs related genes in IGF2 LOI and MOI CRC cells; (**C**) the sphere formation assay in IGF2 LOI and MOI CSCs. After 7 days, IGF2 KD cells showed lower sphere formation efficiency than negative control or blank group (cells without any treatment) in IGF2 LOI CSCs(p<0.05). IGF2 OE cells showed higher sphere formation efficiency than negative control or blank group(p<0.05). Sphere forming efficiency = number of actual spheres/number of cells plated×100.

The IGF2 imprint was detected which separated CRC cells into IGF2 LOI and IGF2 MOI groups which showed Caco2 and HT-29 cells in IGF2 LOI group had a higher methylation in DMRs and IGF2 expression than Hct-8 and Hct-116 cells in IGF2 MOI group (p<0.05, Supplementary Figure 1). In order to explore the relationship between IGF2 LOI and CSCs pluripotency, the CSCs marker CD133 was used in IF and flow cytometry analysis which demonstrated IGF2 LOI cells not only had a higher IGF2 expression but also a higher numbers of CD133^+^ cells compared with MOI cells(p<0.05, Figure 1A). Next CD133 and other CSCs related genes such as CD44, KLF4, SOX2, OCT4 and MYC4 expression were also detected by real-time PCR. Consistent results were found that IGF2 LOI group showed a higher CD133, SOX2, OCT4 and a lower KLF4 expression than IGF2 MOI group(p<0.05, Figure 1B). At last, FACS was applied to isolate CSCs from CRC LOI and MOI cells and sphere-formation assay was performed to further investigate the affection of IGF2 LOI in CSCs pluripotency. The results showed that IGF2 LOI CRC cells usually had a higher CSCs sphere forming efficiency than MOI cells (p<0.05, Figure 1C and Supplementary Figure 2A). Moreover, IGF2 KD was performed in IGF2 LOI group which declared the CSCs sphere forming efficiency significantly decreased since IGF2 expression was silenced. Meanwhile, IGF2 OE was carried out in IGF2 MOI group which increased CSCs sphere forming efficiency was observed due to the up-regulation of IGF2 expression (p<0.05, Figure 1C).

### IGF2 LOI promotes CRC stem cell pluripotency by affecting CSCs autophagy

**Figure 2:**
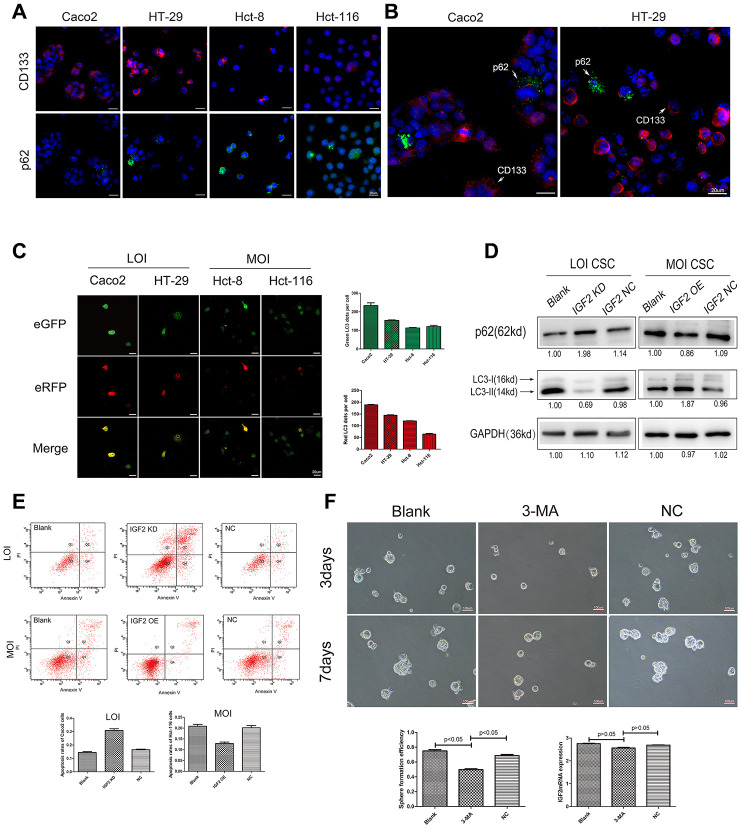
**The affection of IGF2 LOI in CSCs autophagy.** (**A**) the IF results of CD133 and p62 in IGF2 LOI cells (Caco2 and HT-29) and MOI cells (Hct-8 and Hct-116); (**B**) the IF results of p62 in Caco2 or HT-29 CD133^+^cells and CD133^-^ cells. CD133^+^ cells showed a lower p62 expression than CD133^-^ cells; (**C**) the green-puncta and red-puncta numbers of CSCs cells in mRFP-eGFP-LC3fluorescence assay. After hunger for 6 hours, IGF2 LOI CSCs showed higher numbers of green and red LC3 dots per cell than IGF2 MOI cells(p<0.05); (**D**) the p62 and LC3-II expression in IGF2 LOI and MOI CSCs with different treatment; (**E**) the apoptosis rates of IGF2 LOI and MOI CSCs with different treatment; (**F**) the sphere formation efficiency and IGF2 mRNA expression of IGF2 LOI CSCs treated with 3-MA.

Using IF assay, we simultaneously examined the expression of CD133 and p62 in IGF2 LOI and MOI CRC cells which declared that IGF2 LOI cells had a higher CD133 expression and a lower p62 expression than MOI cells (Figure 2A). Interestingly, we found that cells showed a higher CD133 expression usually had a lower p62 expression than other cells, especially in LOI cells (Figure 2B). Therefore, the relationship between IGF2 LOI and CSCs autophagy was further explored. FACS was applied to isolate CSCs from CRC LOI and MOI cells. mRFR-eGFR-LC3fluorescence assay was firstly performed in CSCs which demonstrated CSCs showed IGF2 LOI usually had a higher number of RFP and EGFP puncta than MOI CSCs (p<0.05, Figure 2C). Then IGF2 KD was carried out on Caco2 CSCs which showed IGF2 LOI and IGF2 OE was applied on Hct-116 CSCs which was defined as IGF2 MOI CSCs. After EBSS hunger for 6 hours, IGF2 KD group showed a lower LC3-II and higher p62 expression than negative control in IGF2 LOI CSCs while in IGF2 MOI CSCs, IGF2 OE group showed a higher LC3-II and lower p62 expression than negative control (p<0.05, Figure 2D). On the basis of these findings, the apoptosis was detected in IGF2 LOI and MOI CSCs. The results showed that IGF2 LOI CSCs had a lower apoptosis than MOI CSCs (p<0.05, Figure 1E and supplementary Figure 2B). Moreover, the apoptosis was also detected in IGF2 KD and OE CSCs which showed that IGF2 KD group had a higher apoptosis than negative control in IGF2 LOI CSCs while IGF2 OE group had a lower apoptosis than negative control in IGF2 MOI CSCs(p<0.05, Figure 2E). At last, 3-MA was used to inhibit IGF2 LOI CSCs autophagy. LC3-II was tested to guarantee the success of autophagy inhibition (Supplementary Figure 2C). The results showed that CSCs treated with 3-Methyladenine showed significant lower sphere forming efficiency than negative control though the IGF2 expression did not show any difference (Figure 2F).

### IGF2 promotes CSCs pluripotency by interacting with overexpressed IR-A rather than IGF1R

**Figure 3:**
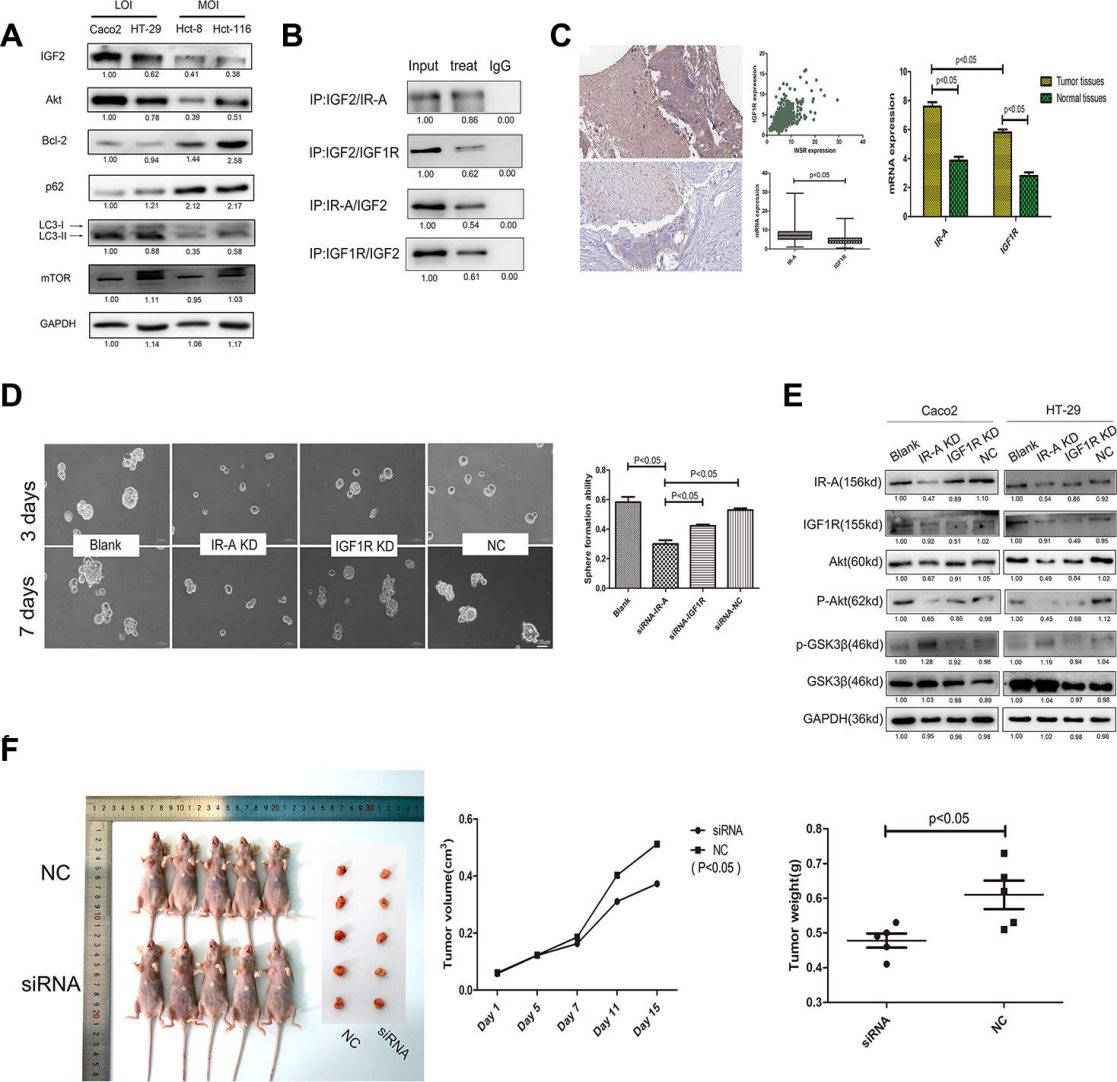
**The potential mechanism of IGF2 in CSCs regulation.** (**A**) the expression levels of potential regulators downstream of IGF2. (**B**) the CO-IP results of IGF2 LOI CSCs with IGF2/IR-A/IGF1R antibodies; (**C**) the IR-A and IGF1R mRNA expression in TCGA and CRC patients; (**D**) the sphere formation efficiency of IGF2 LOI CSCs transfected with IR-A or IGF1R siRNA; (**E**) the changing of Akt, GSK3β expression in IGF2 LOI CSCs transfected with IR-A or IGF1R siRNA; (**F**) Typical pictures and dates of nude mice with tumorigenicity and tumors which were under different treatments.

Western-blot was used to explore the potential downstream regulators of IGF2. We found though significant difference was not detected on the expression of mTOR, IGF2 LOI CSCs showed a higher Akt and lower expression of Bcl-2, an autophagy inhibitor, than IGF2 MOI CSCs (Figure 2F). By CO-IP assay in IGF2 LOI CSCs, we initially examined the proteins interacting with IGF2. As showed in Figure 3A, insulin receptor isoform A (IR-A) and IGF1receptor (IGF1R) both had the ability to interact with IGF2. However, by comparing with input, IR-A expressed higher than IGF1R when IGF2 antibody was used to immunoprecipitate IR-A or IGF1R. Then the expression of IR-A and IGF1R in 438 CRC patients was analyzed from the database TCGA and The Human Protein Atlas. As a result, we found IR-A and IGFIR expression was positively correlated, but IR-A showed a significant higher expression than IGF1R in tumor tissues (p<0.05, Figure 3B). Meanwhile, 20 CRC patients’ tumor and paired normal tissues were also collected and used to detect the IR-A and IGF1R mRNA expression. As a result, both of them showed a higher expression in tumor tissues than normal controls, but IR-A showed a higher expression in tumor tissues than IGF1R (p<0.05, Figure 3C). Next, siRNA of IR-A and IGF1R were transfected to LOI CSCs which IR-A KD group showed the most significant sphere forming efficiency compared with IGF1R KD group and negative control(p<0.05, Figure 3D). Furthermore, CSCs treated with siRNA of IR-A showed a lower Akt, p-Akt and a higher phosphor-GSK3β expression than cells treated with siRNA of IGFIR (p<0.05, Figure 3E). In addition, tumorigenicity assay was examined in mice injected with or without IR-A mRNA siRNA lentivirus. As expected, tumors showed a significant lower IR-A expression in mice injected with IR-A mRNA knockdown lentivirus than negative control (Supplementary Figure 2D). Further results showed that mice with IR-A knock down had a lower tumor volume and weight than negative control (p<0.05, Figure 3F).

### Down-regulated miRNA-195 contributes to IR-A overexpression

**Figure 4:**
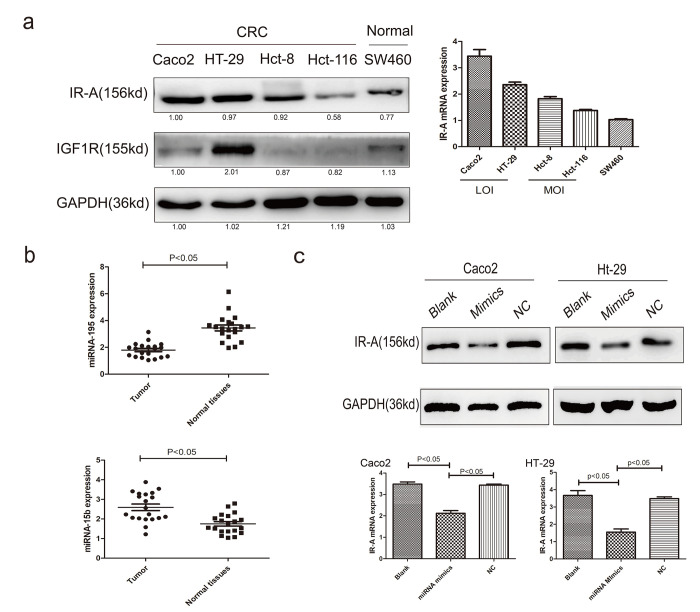
**The regulation of miRNAs on IR-A expression.** (**A**) the IR-A and IGF1R protein and mRNA expression in CRC and normal cells; (**B**) the expression levels of miRNA-195 and miRNA-15b in CRC patients; (**C**) the IR-A expression of IGF2 LOI CRC cells transfected with miRNA-195 mimics.

The correlation of IR-A expression and IGF2 LOI was firstly explored in CRC stem cells which showed that CSCs all showed a higher IR-A expression than normal cells, but significant difference between IGF2 LOI CSCs and MOI CSCs (p<0.05, Figure 4A). Then TargetScan database was used to find potential miRNA on regulating IR-A expressison as described previously [13]. miRNA-195 and miRNA-15b were reported to play an important role on IR-A inhibition. Then real-time PCR was performed on CRC tumor and normal tissues which declared that miRNA-195 showed a significantly lower expression on tumor tissues compared with normal tissues, meanwhile, miRNA-15b showed a higher expression (p<0.05, Figure 4B). Considering the high expression of IR-A on tumor tissues, miRNA-195 mimics was designed and used in the next studies. The Caco2 and HT-29 CSCs transfected with miRNA-195 mimics showed a significantly lower IR-A expression than negative control (p<0.05, Figure 4C).

## DISCUSSION

The existence of CSCs that have the capacity to resist conventional chemo-radiotherapy and to self-renew is one of the major challenges in CRC treatment [14]. Pluripotency is a key feature of CSCs that allows them to indefinitely divide and maintain the undifferentiated state [15]. It was made clear that autophagy is up-regulated in the mammospheres compared to adherent cells which were an intrinsic feature for the maintenance of CSC pluripotency under various pathophysiological conditions [16]. In this study, we fortunately found that IGF2 LOI played an important role in CRC stem cells pluripotency by promoting CSCs autophagy.

IGF2 is often overexpressed in a variety of human malignancies, partially due to the loss of genomic imprinting. Aberrant IGF2 imprinting may be an intrinsic epigenetic control mechanism that enhances stemness, self-renewal and chemo/radiotherapy resistance in cancer stem cells [17]. Stem cells expressed both IGF1R and IR-A, with a predominance of the latter, and to be exquisitely sensitive to IGF2 for cell self-renewal [18, 19]. Recently, it had been showed that the expression of the secreted IGF2 and phosphorylation of IGF1R, were necessary in promoting lung CSCs sphere formation [20]. By the way, the over-activation of IR-A/IGF-2 loop was also reported to be associated with thyroid cancer stem-like features and refractoriness to some targeted therapies [21]. However, the role of IGF2 epigenetic regulation in CRC stem cells has not been elucidated. By examining the IGF2 MDR methylation and allelic expression using an IGF2 exon 9 SNP, we firstly confirmed IGF2 LOI played an important role in CRC stem cell pluripotency. Then IR-A and IGF1R were detected in understanding the potential mechanism of IGF2 in CSCs pluripotency regulation. Both of them showed a high expression in CRC tumor tissues and cells, IR-A seemed to be expressed higher than IGF1R which made IR-A to have a higher ability than IGF1R to interact with IGF2. Therefore, early efforts to target the insulin/IGF signaling axis focused on the IGF1 receptor as a therapeutic target was usually quickly acquired which might be due to the compensatory signaling through the IR-A [22].

Mammalian target of rapamycin (mTOR) is a protein kinase regulating CSCs generation which was reported to be a key factor in inducing cell autophagy [23, 24]. Recent study demonstrated IGF2/IGF1R signal mediated cell autophagy by suppression of the PI3K-Akt-mTOR signaling pathway was also found to play an important role in CRC [25]. Thereby, Akt and mTOR expression were firstly detected in CRC stem cells which Akt showed a higher expressions in IGF2 LOI CSCs but significant difference was not found between IGF2 LOI CSCs and MOI CSCs in mTOR expression. However, the high expression of LC3-II and decreased p62 expression both indicated IGF2 LOI CSCs showed a higher authophagy than MOI CSCs. Rather than IGF2/IGF1R/mTOR signal, other signal seemed to have a more affection in IGF2 LOI CSCs autophagy. Studies showed activation of Bcl-2 as a mediating event played an important role in autophagy inhibition which was stimulated by GSK3β [26, 27]. Moreover, Akt is one of the most extensively studied regulators of GSK3. All three isoforms of Akt (Akt1, Akt2 and Akt3) are recognized to phosphorylate S9 of GSK3β and inactivate it [28]. Fortunately, IGF2 LOI CSCs showed a higher Akt expression which was found to have a lower Bcl-2 expression than MOI CSCs. By mRNA interference, we found IGF2/IR-A signal seemed to play a more important role than IGF2/IGF1R in promoting Akt expression and phosphorylation which further decreased GSK3β phosphorylation.

MicroRNAs can function as negative regulators of target genes by repressing the translation of mRNAs to inhibit protein expression, or directly degrading mRNAs [29]. An increasing number of evidence has also indicated that miRNAs are the key players in maintaining the characteristics of CSCs for self- renewal, proliferation, differentiation and chemoresistance, and they can be used for diagnostic, prognostic and therapeutic targets for the metastasis, drug response and treatment of cancers [30, 31]. IR-A had been suggested to be involved in lots of cancer development [32]. The expression of IR-A was firstly detected in IGF2 LOI CSCs compared with MOI CSCs which unfortunately showed the overexpression of IR-A was not correlated to IGF2 LOI, two microRNAs miRNA-195 and miRNA-15b were selected from TargetScan database which finally found miRNA-195 played an more important role on IR-A expression inhibition. The low miRNA-195 expression on CRC patients promoted the IGF2/IR-A binding which further boosted the CSCs pluripotency.

## MATERIALS AND METHODS

### Cell culture and treatment

Cell lines Caco2, HT-29, Hct-8, Hct-116(human colon cancer cell lines) and FHC (normal human intestinal epithelial cell line) were obtained from Shanghai Cell Collection, Chinese Academy of Sciences. All above cell lines were maintained in DMEM (Hyclone, USA) containing 10% fetal bovine serum (FBS; Hyclone) and cultured at 37°C in a humidified atmosphere with 5% CO2. All the tumor cell lines had been tested by short tandem repeat (STR) method in ABI 3500 Genetic Analyzer (USA Life 3500). EBSS purchased from Life Technologies (14155-063, Gibco, USA) and 3-methyladenine (3-MA, KeyGEN, China) were used in inducing and inhibiting autophagy.

### Sphere-formation assay

In order to form CSCs spheres, 1×10^4^ cells were plate donto poly HEMA (Sigma)-coated 6-well-plates, medium with B27 supplement 20 ng/mL, EGF20 ng/mL, basic fibroblast growth factor and 20 ng/mL in serum free DMEM-F12 (HyClone, UT). The primary CSCs spheres were collected and dissociated into single cells, and then re-suspended in the medium to culture next-generation spheres for 7 days. Then the second generation cells were used in the next experiments. CSC spheres were defined as cell colonies with a diameter >50μm and area >50% showing three-dimensional structure and blurred cell margins. Percentage of sphere forming efficiency was calculated as (number of actual spheres/number of cells plated×100) [33].

### Clinic samples

Tumor tissues and matched adjacent normal tissues were obtained from enrolled CRC patients who underwent surgery at Nanjing First Hospital Affiliated to Nanjing Medical University, between 2018 and 2019. All specimens were immediately frozen in liquid nitrogen after surgery and stored at -80°C. No patients received chemotherapy or radiotherapy at pre-operation. The medical ethics committee of the Nanjing First Hospital Affiliated to Nanjing Medical University approved the study.

### Allelic expression of IGF2

As reported on previous studies [17], exon 9 of the human IGF2 gene is used to evaluate the IGF2 polymorphism on CRC cell lines(Caco2, HT-29, Hct-8 and Hct-116) by PCR which is known as an ApaI digestion single nucleotide polymorphism (rs680). The primer sequences are as follows: IGF2Fw: 5'-CCTTGGACTTTGAGTCAAATT-3' and Rev: 5'-GGTCGT GCCAATTACATTTCA-3'. The PCR conditions were initial denaturation for 5 min at 94 ºC, followed by 35 PCR cycles of denaturing at 94ºC for 30s, annealing at 55ºC for 30 s, and elongation at 72ºC for 30s. The RT-PCR products were also digested by ApaI and the amplified products were sequenced. Cells that maintain normal imprinting express a single parental allele, while the LOI showed biallelic expression of IGF2.

### Methylation analysis of IGF2 DMR

Genomic DNA from informative samples was treated with bisulfite (EpiTect Plus DNA Bisulfite Kit 59124) to convert unmethylated cytosines to uracils, whereas methylated cytosines were unaffected. The region corresponded to GenBank were listed in Supplementary Table 1. The PCR condition was as follows: denatured at 95°C for 15min, followed by 95°C for 30sec, 57°C for 30sec and 72°C for 20sec respectively for 50 cycles, and elongated at 72°C for 5 min. Each PCR product (5ul) was analyzed on 2% agarose gel and the remaining 20ul were purified using QIAquick purification kit (Qiagen) to be sequenced using the forward primer, and the sequencing results were analyzed using CpG viewer, a software tool for DNA methylation available by free download from http://dna.leeds.ac.uk/cpgviewer/download.php.

### Reverse transcriptase-quantitative PCR

Total RNA were extracted simultaneously from paraffin-embedded tissues using E.Z.N.A.FFPE RNA Kit (omega, USA) following the manufacturers' protocol. To avoid the possible DNA contamination, RNA was digested with RNase free DNaseI and cleaned with the RNeasy MinElute Cleanup Kit (Qiagen). The primers of IGF2, H19, CD133, CD44, KLF4, SOX4, OCT4, MYC were showed in Supplementary Table 1. Quantitative PCR was performed in duplicates using the Takra qPCR Master Mix in ABI Prism 7700 SDS. Results in all figures show mean ± standard error.

### Flow cytometry analysis and isolation of CRC stem cells

As previously reported [34], expression of the CSC marker CD133 was finally used to detect and isolate CSCs from CRC. Briefly, non-specific binding of cell membranes was blocked by incubating with 5% BSA for 30 min at room temperature. Cells were then incubated with CD133 antibody (MACS, Miltenyi Biotec, Germany) for 30 min before being subjected to flow cytometry analysis (FACSAria, BD, CA). After being washed with phosphate-buffered saline (PBS), ten thousand events per sample were acquired and the cells were counted and collected which were designated as CD133^+^ cells (CSCs) and CD133^−^ cells(non-CSCs).

### Immunofluorescence(IF)

Cells were plated onto sterile cover slips at a concentration of 5×10^5^ cells in fully supplemented keratinocyte media and allowed to adhere for approximately 4h. Cells were then washed twice with PBS and fixed with 10% neutral-buffered formalin for an hour at room temperature. Cover slips with fixed cells were incubated overnight at 41°C with antibodies (1:50, Supplementary Table 1). FITC and Rhodamine B-conjugated anti-rabbit secondary antibody (1:100; Sigma, Canada) was used for detection and cells were counterstained with propidium iodide. Nuclear staining was with DAPI (Molecular Probe) diluted1:1000 and incubated at RT for 30 min. After washing with PBS, the cells were dyed green or red fluorescent and examined by fluorescence microscopy (Axioplan, Zeiss).

### Transfection and establishment of cell lines

Three different specific siRNAs of IGF2, IR-A and IGF1R were designed and formed (Genepharma, China). Following manufacturer’s protocol (Genepharma, China), transfection reagent was used for transient transfection in cells. Then the siRNA of each gene which showed most inhibition efficiency was used in the next experiments. The list of siRNAs and negative control were showed in Supplementary Table 1. In order to overexpressed IGF2 mRNA in cells. The synthetic IGF2 DNA fragment and lentiviral vector PGMLV-6395 were enzyme digested with BamH I/EcoRI, Ligation was performed to construct IGF2 overexpressed lentivirus recombinant plasmid. The following lentivirus package was performed by Auragene Biotech (Changsha, China). In order to overexpressed miRNA in cells, miRNA mimics was formed (Genepharma, China), and transfected with lip2000(Invitrogen, USA).

### CO-Immunoprecipitation (CO-IP)

Following cell lysis by RIPA lysis solution, supplemented with protease and phosphatase inhibitors, proteins were obtained by centrifugation at 4 °C. The protein concentration was established using a BCA kit. Equivalent quantities of protein were incubated with an antibody overnight at 4 °C. The next day, they were incubated with Protein G/A agarose beads on a horizontal rotator. Beads-antibodies compound was harvested and freed out the antigen and antibody for Western blot analysis with antibodies against IR-A and IGF1R.

### Western blot analysis

Samples were lysed using a lysis buffer. Then, the protein concentration was quantified using a BCA protein assay kit (Beyotime, Shanghai, China). Sodium dodecyl sulfate-polyacrylamide gel electrophoresis and Western blot analyses were performed according to the standard procedures. Primary antibodies used were showed in Supplementary Table 1. Secondary antibodies were goat anti-rabbit HRP secondary antibody (System Biosciences, Mountain View, CA). Image J was used to detect the gray value of each strip.

### mRFP-eGFP-LC3 fluorescence assay

Cells with mRFR-eGFR-LC3 constitutive expression were treated with earle's balanced salts solution (EBSS) purchased from Life Technologies (14155-063, Gibco) for 6 hours. Then fixed with 4% PFA for 30 min and washed with PBS for 3 times. The immunofluorescent images were obtained by using a confocal laser scanning microscope (Leica TCS SP8, Solms, Germany) and typical images were presented. For quantitative assay, the green-puncta and red-puncta numbers were counted

### Apoptosis assay

AnnexinV-FITC/PI kit (Keygentec, China) was used in this study. According to the kit instructions, Cells were digested using 0.1% trypsin without EDTA and centrifuged at 1000 rpm. Then binding buffer was used to suspend cells, keeping cell concentration at 5 × 10^5^ cells/mL. Annexin V and PI were added, and the mixture was further incubated for 15 min. After the incubation, cell apoptosis was detected using flow cytometry within 1h. In the results with 4 quadrants, the fourth quadrant represented the number of early apoptotic cells, and the apoptosis rate was calculated.

### Tumorigenicity in nude mice

Animal experiments were performed in strict accordance with the guide for the Care and Use of Laboratory Animals of the Nanjing First Hospital Affiliated to Nanjing Medical University. 20 Female BALB/c nude mice aged 6-8 weeks were used. Then CD133^+^ cells were resuspended in serum-free medium and mixed with matrigel at the ratio of 1:1. NOD/SCID mice were randomly divided into 2 groups. 1×10^5^ indicated cells were inoculated subcutaneously into the inguinal folds of NOD/SCID mice. 7 days after inoculation, tumor formation was evaluated by palpation of injection sites, then the mice that developed palpable tumors were injected with or without IR-A mRNA knockdown lentivirus at the injection site every 3 days for 3 times. At the end of experiment (21 days), the mice were sacrificed under deep anesthesia with pentobarbital and eosin (HE) staining. The tumors were then dissected and captured.

### Computational analysis of TCGA RNA-Seq datasets

We downloaded the CRC RNA-Seq datasets (Illumina HiSeq2000) from The Cancer Genomics Atlas (TCGA) and The Human Protein Atlas, processed and analyzed using R statistical software package “ggplot2”.

### Statistical analysis

Results from at least three independent experiments were expressed as mean ± SD. Statistical analysis of data from two groups was compared by two-tail t-test. Data from multiple groups was performed by one-way ANOVA, followed by Tukey post test. Statistical significance was determined as P < 0.05.

## CONCLUSION

In conclusion, the present study explored the potential relationship and mechanism of IGF2 LOI in CRC stem cells development which finally demonstrated IGF2 LOI promoted CRC stem cells pluripotency by promoting CSCs autophagy. For the degradation of miRNA-195, IGF2 showed a higher ability in interacting with overexpressed IR-A rather than IGF1R which further modulated CSCs autophagy might provide new mechanistic insight into the CRC diagnose and treatment.

## Supplementary Material

Supplementary Table 1

Supplementary Figures
