# Supplementary material for: IGF2 loss of imprinting enhances colorectal cancer stem cells pluripotency by promoting tumor autophagy
Source: Aging (Albany NY). 2020 Nov 5;12(21):21236–52. doi: 10.18632/aging.103837 (PMC7695407; doi:10.18632/aging.103837)
Supplement: Supplementary Table 1 [file aging-12-103837-s001..pdf]

SUPPLEMENTARY TABLE

Supplementary Table 1. The information of all the primers or antibodies used in the study.

**Methylation sites**	
IGF2 DMR0	GenBank nucleotides 631–859, accession No. Y13633
IGF2 DMR1	GenBank nucleotides 155440-155238, accession No. AC130303
IGF2 DMR2	GenBank nucleotides 155737-155911, accession No. AC130303
H19 ICR	GenBank nucleotides 7881–8100 accession no. AF125183
Real-time PCR primers	
IGF2	F: 5'- CCTTGGACTTTGAGTCAAATT -3'
	R: 5'- GGTCGT GCCAATTACATTTCA -3'
H19	F: 5'- TTACTTCCTCCACGGAGTCG-3'
	F: 5'- GAGCTGGGTAGCACCATTTC-3'
GAPDH	F: 5'-GTCAACGGATTTGGTCGTATT-3'
	R: 5'-AGTCTTCTGGGTGGCAGTGAT-3'
CD133	F: 5'-ACCCAAGACTCCCATAAAGC-3'
	R: 5'-TACAAAAGAAATACCCCACC-3'
CD44	F:5' - GAGCATCGGATTTGAGA -3'
	R: 5'- CATACTGGGAGGTGTTGG -3'
KLF4	F: 5'-CCGCCGCTCCATTACCAA-3'
	R: 5'-CATCCACAGCCGTCCCAG-3'
SOX2	F: 5'-ACCAGCGCATGGACAGTTAC-3'
	R: 5'-CCGTTCATGTAGGTCTGCGA -3'
OCT4	F: 5'-AGAAGCTGGAGCAAAACCCG-3'
	R: 5'-ACCTTCCCAAATAGAACCCCCA-3'
MYC	F: 5'-ACGAAACTTTGCCCATAGC-3'
	R: 5'-CGAGGTCATAGTTCCTGTTG-3'
IR-A	F: 5'-GTTTTCGTCCCCAGGCCAT-3'
	R: 5'- CCACCGTCACATTCCCAAC -3'
IGF1R	F: 5'- GTCCAGGCCAAAACAGGATA -3'
	R: 5'- CAGAGGCATACAGCACTCCA -3'
IF Antibodies	
IGF2	ab9574, Abcam, UK
p62	ab211324, Abcam, UK
CD133	ab19898, Abcam, UK

Western-blot Antibodies	
IGF2	ab170304, Abcam, UK
CD133	ab19898, Abcam, UK
p62	16177, CST, USA
LC3	3868, CST, USA
Akt	10176-2-AP, Proteintech, China
p-Akt	10176-2-AP, Proteintech, China
GSK3β	12456T, CST, USA
p- GSK3β	9322S, CST, USA
Bcl-2	ab182858, Abcam, UK
mTOR	ab2732, Abcam, UK
IR-A	ab5500, Abcam, UK
IGF1R	ab39675, Abcam, UK
GAPDH	10494-1-AP, Proteintech, China
RNA silence	
IGF2	F: 5'-GCAAGUUCUUCCAAUAUGATT-3'
	R: 5'-UCAUAUUGGAAGAACUUGCTT-3'
IR-A	F: 5'-GCAUGGAUAUCCGGAACAATT-3'
	R: 5'-UUGUUCCGGAUAUCCAUGCTT-3'
IGF1R	F: 5'-GCACAAUUACUGCUCCAAATT-3'
	R: 5'-UUUGGAGCAGUAAUUGUGCTT-3'
Negative control	5'-GGAUUUCGAGUCGUCUUAAUGUAUA-3'
