# Supplementary material for: IGF2 loss of imprinting enhances colorectal cancer stem cells pluripotency by promoting tumor autophagy
Source: Aging (Albany NY). 2020 Nov 5;12(21):21236–52. doi: 10.18632/aging.103837 (PMC7695407; doi:10.18632/aging.103837)
Supplement: Supplementary Figures [file aging-12-103837-s002..pdf]

SUPPLEMENTARY FIGURES

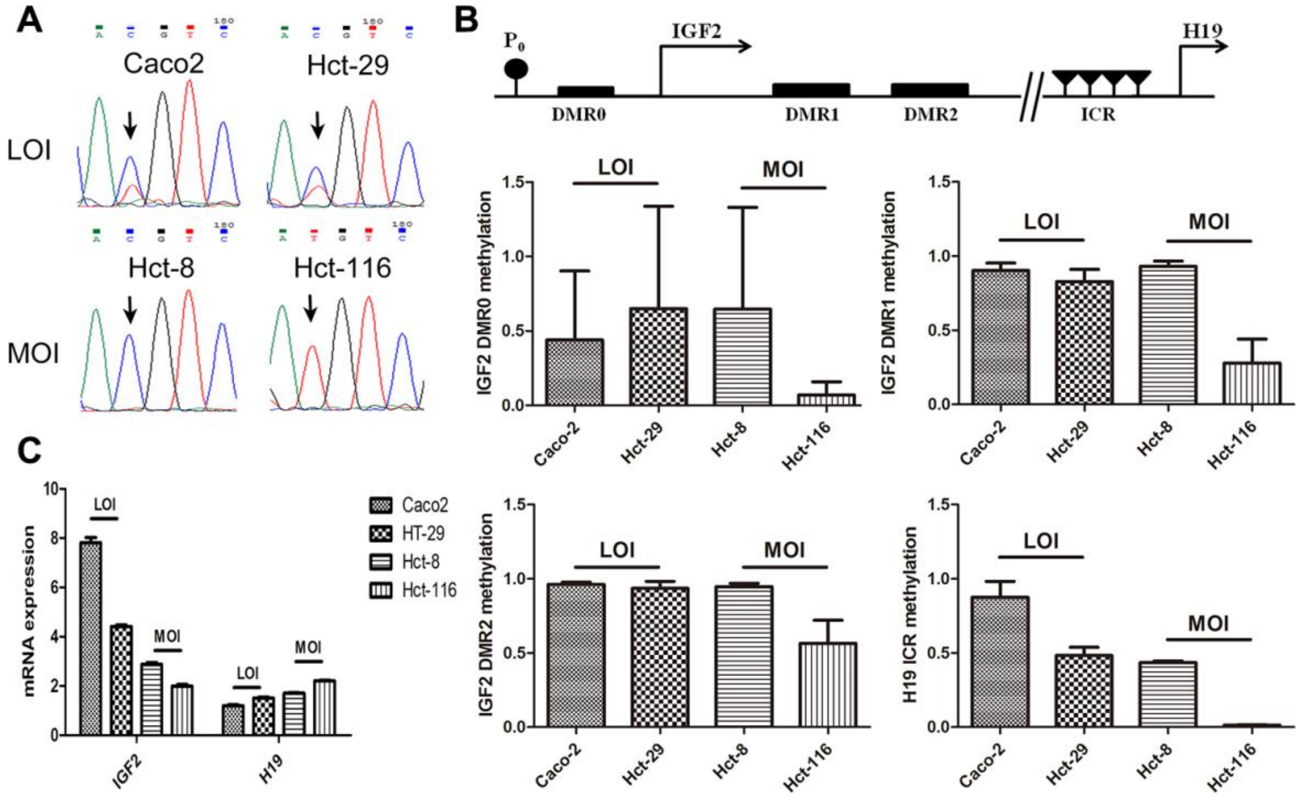

Supplementary Figure 1. The detection of IGF2 imprint status and expression in CRC cells. (**A**) the sequencing results of IGF2 rs680 single nucleotide polymorphism; (**B**) the results of methylation analysis in IGF2 DMR; (**C**) the expression levels of IGF2 and H19 in CRC cells.

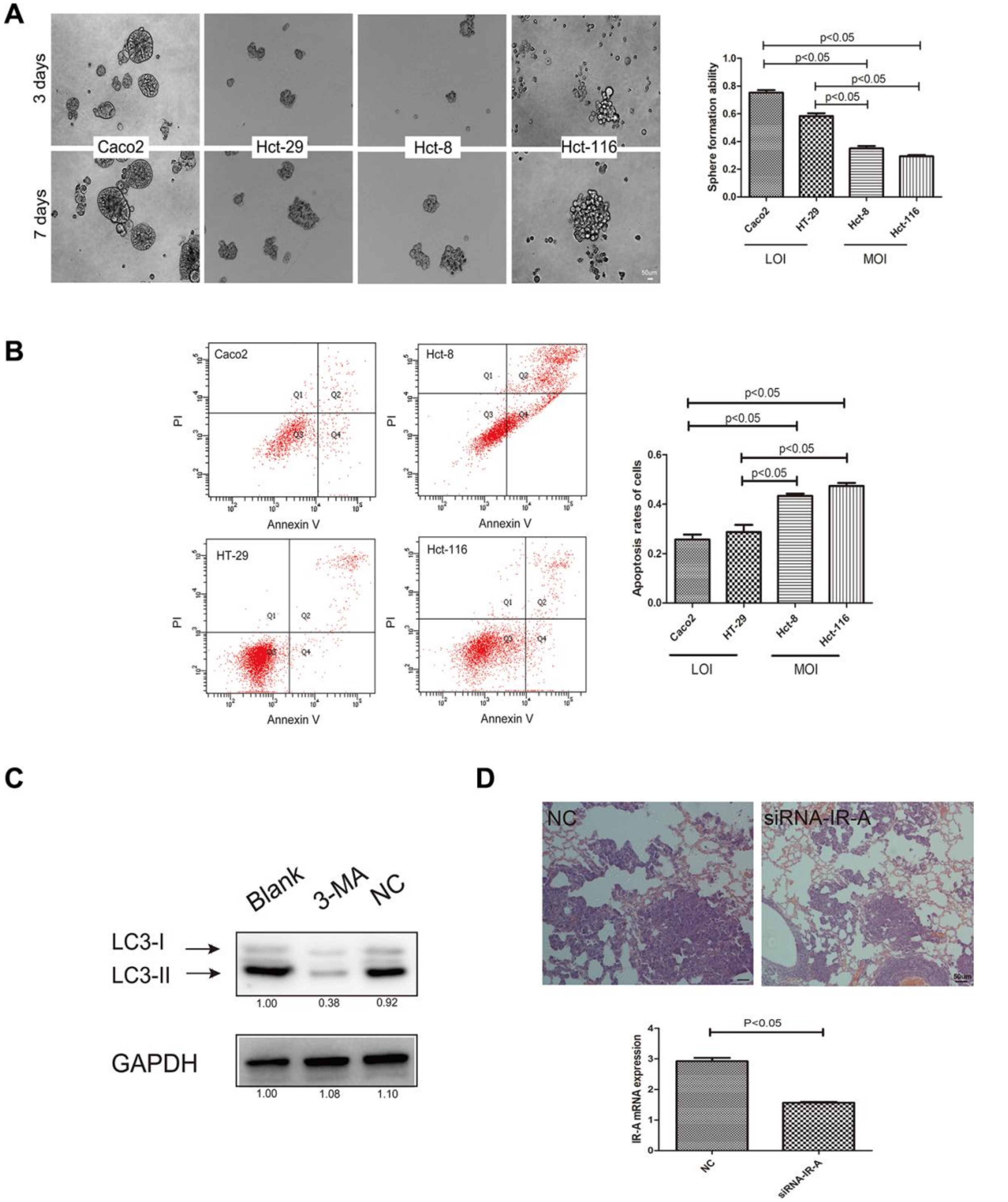

Supplementary Figure 2. The biological differences of CRC cells with or without treatments. (**A**) the sphere forming results of CRC cells with or without IGF2 LOI; (**B**) the comparison of apoptosis rates between IGF2 LOI and MOI CRC cells; (**C**) the LC3-II expression levels of IGF2 LOI CSCs with or without 3-MA treatment; (**D**) the HE and IR-A expression results of mice transfected with IR-A mRNA siRNA lentivirus.

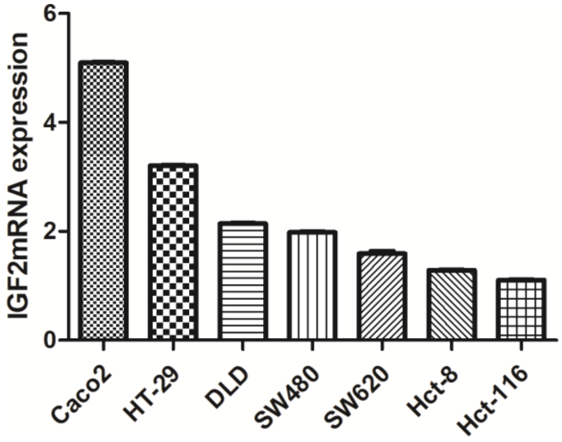

Supplementary Figure 3. The IGF2 expression on different CRC cell lines.
